# Cooperative Substructure and Energetics of Allosteric Regulation of the Catalytic Core of the E3 Ubiquitin Ligase Parkin by Phosphorylated Ubiquitin

**DOI:** 10.3390/biom14101338

**Published:** 2024-10-21

**Authors:** Xiang Ye, Sravya Kotaru, Rosana Lopes, Shannen Cravens, Mauricio Lasagna, A. Joshua Wand

**Affiliations:** 1 Department of Biochemistry & Biophysics, Texas A&M University, College Station, TX 77843, USA; 2 Department of Biochemistry & Biophysics, Perelman School of Medicine, University of Pennsylvania, Philadelphia, PA 19104, USA; 3 Department of Chemistry and Biochemistry, Gonzaga University, Spokane, WA 99258, USA; 4 Department of Chemistry, Texas A&M University, College Station, TX 77843, USA

**Keywords:** Parkin E3 ubiquitin ligase, hydrogen exchange, allostery, protein stability, protein ensemble

## Abstract

Mutations in the parkin gene product Parkin give rise to autosomal recessive juvenile parkinsonism. Parkin is an E3 ubiquitin ligase that is a critical participant in the process of mitophagy. Parkin has a complex structure that integrates several allosteric signals to maintain precise control of its catalytic activity. Though its allosterically controlled structural reorganization has been extensively characterized by crystallography, the energetics and mechanisms of allosteric regulation of Parkin are much less well understood. Allostery is fundamentally linked to the energetics of the cooperative (sub)structure of the protein. Herein, we examine the mechanism of allosteric activation by phosphorylated ubiquitin binding to the enzymatic core of Parkin, which lacks the antagonistic Ubl domain. In this way, the allosteric effects of the agonist phosphorylated ubiquitin can be isolated. Using native-state hydrogen exchange monitored by mass spectrometry, we find that the five structural domains of the core of Parkin are energetically distinct. Nevertheless, association of phosphorylated ubiquitin destabilizes structural elements that bind the ubiquitin-like domain antagonist while promoting the dissociation of the catalytic domain and energetically poises the protein for transition to the fully activated structure.

## 1. Introduction

**Figure 1:**
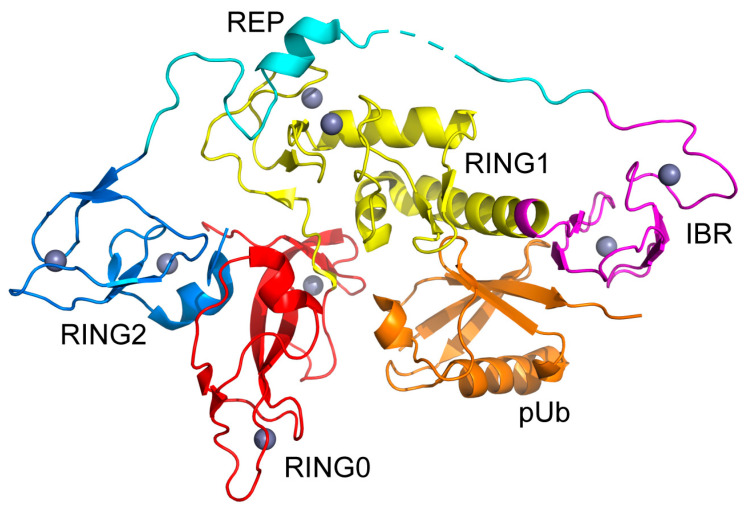
Cartoon representation of the structure of the Parkin(∆Ubl)·pUb complex. RING0 (residues 142–225, red), RING1 (residues 226–329, yellow), IBR (residues 330–378, magenta), REP (residues 379–410, cyan), RING2 (residues 411–465, blue), zinc atoms (wheat) and pUb (orange). Drawn using PyMol from the structure determined by Wauer et al. (PDB code: 5caw) [10].

Parkin is an E3 ubiquitin ligase specifically involved in the disposal of damaged mitochondria via controlled autophagy (also known as mitophagy). Mitophagy has a central role in human physiology, and its disruption is a causative feature of numerous human diseases [1]. Autosomal recessive mutations in the gene coding for Parkin [2] lead to enzymatic dysfunction or uncontrolled proteostasis [3,4]. Inadequate regulation of Parkin activity often results in uncontrolled mitophagy and has been associated with early-onset Parkinson’s disease (eoPD) [2,4,5,6,7,8]. Parkin has a complex multidomain structure that integrates several allosteric regulatory elements. The core of Parkin, defined here to include residues 137–461 in the human protein, which we refer to as Parkin(∆Ubl), exhibits the so-called RING-between-RING architecture (Figure 1). It has five domains: RING0 (residues 142–225), RING1 (residues 226–329), IBR (residues 330–378), REP (residues 379–410) and RING2 (residues 411–465). RING2 contains the active site Cys_431_. RING1 recognizes and binds ubiquitin-charged E2 enzymes, and REP is thought to interfere with this interaction in the repressed state. RING0 serves to block the active site Cys in the repressed state. Parkin binds eight zinc ions using zinc fingers in the IBR, RING0, RING1 and RING2 domains [9].

The first ~140 residues of Parkin contain a ubiquitin-like domain (Ubl) attached to the core with a largely disordered linker [9]. Most crystallographic studies of Parkin have stitched the Ubl domain to the RING0 domain of the core. An AlphaFold analysis of the human protein’s primary sequence does not predict structured elements in the linker region [11,12]. The structure of full-length Parkin bound to phosphorylated ubiquitin indicated a small helical segment contained within a structured element of the linker (residues 101–110) [13].

Parkin is exquisitely regulated through the action of both phosphorylation and by binding of the Ubl domain in *cis* and the binding of phosphorylated ubiquitin (pUb) in *trans* (reviewed by Trempe and Gehring [14]). Parkin undergoes extensive structural reorganization upon activation to reveal the active-site Cys residue of the RING2 domain [13]. Although the structural transitions underlying the allosteric regulation of Parkin by its tethered ubiquitin-like domain (Ubl) and phosphorylated ubiquitin (pUb) are reasonably well characterized, the structures do not reveal the energetics and dynamics of Parkin regulation. Very little is known quantitatively about how the opposing effects of Parkin’s interaction with its tethered Ubl domain and pUb combine with phosphorylation of Parkin to regulate its activity. Herein, we isolate and explore the energetics of the interaction of pUb with the Parkin core using the native-state hydrogen exchange approach [15]. Various aspects of Parkin structural transitions have been examined by qualitative hydrogen exchange relying on changes in protection of otherwise solvent-exposed amide hydrogens to map changes in the topology of the protein. Herein, the native-state HX methodology is used to reveal the energetic coupling of pUb binding to the various domains of Parkin(∆Ubl).

## 2. Materials and Methods

### 2.1. Protein Expression and Purification

A gene for the Parkin(∆Ubl) codon optimized for expression in *E. coli* and corresponding to (SS) followed by residues 135 through 465 of the canonical isoform (Swiss-Prot O60260-1; https://www.uniprot.org/; accessed on 16 May 2018) of human Parkin was prepared in a His-SUMO tag pET32b vector (Genscript, Piscataway, NJ, USA). Expression was carried out in BL21 (DE3) *E. coli* during growth on M9 media. The His-SUMO tag Parkin fusion protein was isolated from inclusion bodies, solubilized in 7 M Gdn·HCl in 50 mM Tris buffer (pH 8) with 5 mM DTT. The fusion protein was refolded by drip dilution into 440 mM arginine, 55 mM Tris (pH 8.2), 21 mM NaCl, 0.88 mM KCl, 0.1 mM ZnCl_2_ and 40 mM DTT. The fusion protein was equilibrated with 50 mM Tris (pH 7.8), 50 mM NaCl and fresh DTT (2 mM) and purified by anion exchange chromatography on a HiTrap Q HP column with a gradient from 0.050 to 1 M NaCl. The fusion protein was then cut by adding TEV protease (1:10 molar ratio) and dialyzed against 50 mM Tris (pH 7.8), 50 mM NaCl and 2 mM DTT. Parkin(∆Ubl) was then purified by size-exclusion chromatography on Superdex 200 (Cytiva, Marlborough, MA, USA) into 50 mM Tris (pH 7.8), 100 mM NaCl and 20% (*v*/*v*) glycerol and 5 mM DTT for long-term storage at −80 °C. Phosphorylated ubiquitin was prepared as described [16].

### 2.2. Protein Stability Measurements

Denaturant unfolding of Parkin(∆Ubl) was followed using a Jasco 1500 CD spectrophotometer (Jasco, Easton, MD, USA). A 10 M stock solution of urea was prepared in water. From this stock, an 8.8 M urea was prepared in 20 mM HEPES, 100 mM NaCl, 1 mM TCEP and 10 μM ZnCl_2_ pH 7.4. A series of urea dilutions from 0 M to 8 M, in 0.5 M increments, were prepared, each containing 11 μM ∆Ubl–Parkin. Samples were aged for 48 h. CD spectra were collected between 210 and 260 nm at 25 °C. The average was obtained from 3 to 4 spectra recorded for every sample using the following parameters: 0.2 nm step, 1 nm slit, 1–2 s integration time and 50 nm/min speed. For the melting curve, the ellipticity (mdeg) at 222 nm was fitted as a function of urea concentration according to the two-state formalism of Santoro and Bolen [17], but with the following corrected equation [18]:

(1) $$Y\left(\left[urea\right]\right)=\frac{Y_{f}+m_{f}\left[urea\right]+\left(Y_{u}+m_{u}\left[urea\right]\right)exp\left(-\frac{∆G_{unf}\left(H_{2}O\right)}{RT}+\frac{m\left[urea\right]}{RT}\right)}{1+exp\left(-\frac{∆G_{unf}\left(H_{2}O\right)}{RT}+\frac{m\left[urea\right]}{RT}\right)}$$

where *Y*([*urea*]) is the measured molar ellipticity at some urea concentration; *Y_f_* and *Y_u_* are the intercepts; *m_f_* and *m_u_* are the slopes of the pre- and post-transition regions and ∆*G_unf_*(*H*_2_*O*) and m for the transition region, respectively.

### 2.3. Hydrogen Exchange Data Collection

Hydrogen exchange (HX) experiments were carried out in *H*_2_*O* or *D*_2_*O* buffers containing 20 mM HEPES, 150 mM NaCl, 10 μM ZnCl_2_, 1 mM TCEP and 2.5% (*v*/*v*) glycerol, with the direct pH reading adjusted to 7.0. When used, urea was at the same concentration in both buffers. Deuterated urea was used to prepare the *D*_2_*O* buffer. Parkin(∆Ubl) or the Parkin(∆Ubl)·pUb complex was incubated with urea overnight at room temperature prior to HX-MS measurements. HX reactions were initiated by dilution into *D*_2_*O* buffer to a final deuteron fraction of 0.91. The final Parkin(∆Ubl) concentration was ~7 μM. If included, the final concentration of pUb was ~12 μM pUb. All HX labeling reactions were carried out at 20 °C and were quenched with 8 M Gnd·HCl and 50 mM glycine adjusted to a pH between 2.45 and 2.50. The quenched reactions were incubated on ice for 30 s before mixing in a 2.5-fold volume of 0.028% TFA, pH = 2.45, to reduce the final Gnd·HCl concentration to less than 2 M, and they were loaded into an integrated liquid chromatography system with the temperature controlled at 0 °C. On-column digestion by pepsin and subsequent crude separation of the peptides by reverse-phase chromatography before mass spectrometry (MS) measurements was carried out as described by Mayne et al. [19]. Peptides were separated with a shaped gradient of acetonitrile from 10% to 40% on a C18 column and directly injected into the mass spectrometer. Mass spectrometry was carried out with an Agilent QTOF 6545XT (Agilent Scientific Instruments, Santa Clara, CA, USA) running in the extended dynamic mode at a small *m*/*z* range from 300 to 1700. The sheath gas flow and temperature were set at 3.5 L/min and 250 °C, respectively. The drying gas was set at 3.3 L/min and 250 °C.

The reproducibility of HX mass(t) curves was determined by comparing fitted centroid *m*/*z* centers between two independent experiments in the absence of urea. Pairwise comparisons of peptide centroids at ten hydrogen exchange time points gave an average difference and root mean square error of 0.003 and 0.018 amu, respectively. The full distribution is provided in Figure S1. Given this very high precision, single samplings at many HX time points, which maximizes the ability to distinguish fitting models, was indicated. In the production runs reported here, HX was sampled at 0.17, 0.5, 1.5, 4.5, 15, 45, 135, 405, 1200, 4320 and 12,960 min for Parkin(∆Ubl) and the Parkin(∆Ubl)·pUb complex and 0.17, 0.5, 1.5, 4.5, 15 and 45 min for free pUb.

### 2.4. Hydrogen Exchange Data Analysis

Deuterated peptides were searched and identified using the software package ExMS2 [20]. Peptides were chosen for subsequent analysis based on (i) being consistently identified among most of the time points and conditions and (ii) being consistent with overlapping peptides. All peptides used in the analysis satisfied the quality criteria described by Kan et al. [20]. Centroids were fitted by ExMS2 from peptide isotopic distributions and corrected for back exchange occurring during preparation for proteolysis by pepsin, reverse-phase chromatography and MS measurement by using an “all D” control. The “all D” measurement was carried out in solution of the same fraction of solvent deuteron at pH 2.0 and equilibrated for one week.

HX centroid time trajectories were fitted to a single exponential curve (Equation (2)), a single stretched exponential curve (Equation (3)), or a double exponential curve (Equation (4)), as statistically warranted, to extract the HX rate(s) averaged at the peptide level:

(2) $$C\left(t\right)=\left[C\left(\infty \right)-C(0)\right]e^{-k_{HX}t}+A$$

(3) $$C\left(t\right)=\left[C\left(\infty \right)-C(0)\right]e^{-{\left(k_{HX}t\right)}^{\beta }}+A$$

(4) $$C\left(t\right)=\left[C\left(\infty \right)-C(0)\right]\left[f_{f}e^{-k_{HX}^{f}t}+f_{s}e^{-k_{HX}^{s}t}\right]+A$$

where *C(t)* is the centroid mass as a function of hydrogen exchange time (*t*); *C*(0) and *C(∞)* are the initial and limiting centroid masses; *β* is the stretch factor; $k_{HX}$, $k_{HX}^{f}$ and $k_{HX}^{s}$ are the desired effective hydrogen exchange rates with superscripts *f* and *s* corresponding to resolved fast and slow rates and A is a constant offset. HX rates were converted to protection factors by using the intrinsic sequence-corrected unprotected chemical rate of exchange [21] averaged over the backbone amide sites of a given peptide. Apparent free energies (${∆G}_{HX}$) were then calculated from the obtained results as described in the main text.

The sensitivity of hydrogen exchange to denaturant urea concentration was characterized using one or two effective structural transitions, each with an effective free energy in the absence of denaturant (${∆G}_{HX}^{0}$) and denaturant dependence (*m*), i.e.,

(5) $$∆G_{HX}\left(\left[urea\right]\right)=RT\;ln\left[e^{-\frac{{∆G}_{1}^{0}+m_{1}\left[urea\right]}{RT}}+e^{-\frac{{∆G}_{2}^{0}+m_{2}\left[urea\right]}{RT}}\right]$$

where ${∆G}_{1}^{0}$ and ${∆G}_{2}^{0}$ are the free energies of the structural transitions leading to deprotection and subsequent hydrogen exchange in the absence of denaturant and $m_{1}$ and $m_{2}$ are the corresponding denaturant dependencies of the structural transition. In some cases, only one structural transition is statistically warranted (e.g., global unfolding) and/or one urea dependence term is effectively zero (e.g., local unfolding). Fitting and statistical analysis were carried out with GraphPad Prism 10.2.3 (GraphPad Software, Boston, MA, USA) and local Python (version 3.11.5) scripts utilizing NumPy (version 1.24.3). Molecular images were generated using the PyMOL Molecular Graphics System, Version 3.0 Schrödinger, LLC (Boston, MA, USA). Some calculations of physical parameters of protein structures were performed using the PDBePISA server (version 1.34) [22].

## 3. Results

It is well established that exchange of amide hydrogens of the polypeptide chain with those of solvent water requires direct exposure to solvent water [23,24]. Within a structured protein, exposure generally results from a superposition of structural transitions often termed “local,” “subglobal” and “global” unfoldings to reflect the degree of exposure of accessible surface area [15]. As a result, simple hydrogen exchange profiles can usually only reliably inform on gross changes in structure that lead to (de)protection of amide hydrogens. Such studies have proven quite useful in illuminating the large structural reorganizations that accompany (in)activation of Parkin [13,25]. Herein, we wish to reveal the energetics of allosteric regulation by the activating effector pUb through its interaction with Parkin(∆Ubl). Deconvolution of the multiple structural transitions that may contribute to the hydrogen exchange of a given amide hydrogen can often be achieved through the so-called native-state hydrogen exchange approach [15]. In the native-state HX method, structural transitions are distinguished by their relative sensitivity to perturbations such as temperature [15], pressure [26,27] or a chemical denaturant [15]. Herein, we use a chemical denaturant (urea) to differentiate various contributions to observed hydrogen exchange rates. Analogous to the linear extrapolation method used to determine protein stabilities [18], the native-state HX method relies on differences in accessible surface area to distinguish structural transitions contributing to hydrogen exchange [15].

### 3.1. Parkin(∆Ubl) Has Low Structural Cooperativity

**Figure 2:**
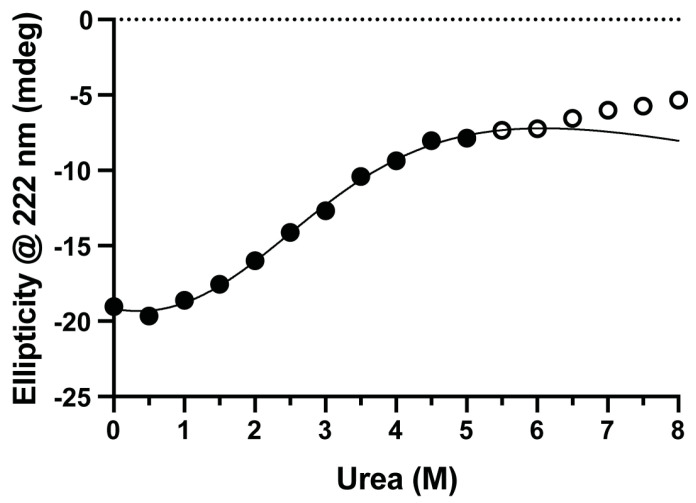
Parkin(∆Ubl) denaturation by urea followed by circular dichroism at 222 nm. The solid line results from fitting the solid circles to Equation (1). For reasons outlined in the main text, the fitted ∆*G_unf_* and m values indicate a relatively uncooperative multistate unfolding of the protein, which is consistent with the hydrogen exchange studies described below. The hollow circles correspond to a separate transition likely involving unfolding of structure stabilized by high-affinity zinc fingers. See main text.

To place the hydrogen-exchange-derived free energy profiles of Parkin(∆Ubl) and its response to the binding of pUb in context, the structural stability and cooperativity of the unfolding of the secondary structure of Parkin(∆Ubl) in response to an increasing urea concentration were observed using circular dichroism spectroscopy (Figure 2). The ellipticity at 222 nm was used to quantify the unfolding of the protein [28]. The unfolding profile of Parkin(∆Ubl) is somewhat globally uncooperative, with two apparent structural transitions: a main one centered around ~3 M and a weaker ellipticity change centered at ~7 M urea. We attribute the former to the transition of Parkin(∆Ubl) to a state that is largely unfolded save for residual structure retained in the eight zinc fingers, which are then unfolded at higher denaturant concentrations. Direct fitting [18] of the data derived from 0 to 5 M urea to a two-state unfolding model results in ill-defined precision for both ∆*G_unf_* and m-value. This is indicative of fitting a two-state model to a low-cooperativity multi-state unfolding equilibrium. It is important to note that this clearly indicates that a simple two-state model for global unfolding of Parkin(∆Ubl) is *inappropriate*. This is reinforced by the hydrogen exchange studies reported below.

### 3.2. HX Indicates That Parkin(∆Ubl) Exists as an Ensemble of Cooperative Units

Hydrogen exchange at pH 7.0 and 20 °C was monitored by peptide fragmentation mass spectrometry, carried out at seven urea concentrations ranging up to 4 M and sampled at eleven time points from 0.125 min to 216 h. Fragmentation by pepsin resulted in over 550 peptides from Parkin(∆Ubl) and over 90 peptides from pUb that were sufficiently resolved by reverse-phase chromatography to be identified and quantitated by mass spectrometry (Figures S1–S4). The average lengths of peptides derived from Parkin(∆Ubl) and pUb were 18 ± 9 and 22 ± 10 residues, respectively. Parkin(∆Ubl) is somewhat resistant to broad digestion by pepsin, and though a high number of peptides are generated, they are somewhat redundant, albeit helpful for providing confidence in peptide identification.

**Figure 3:**
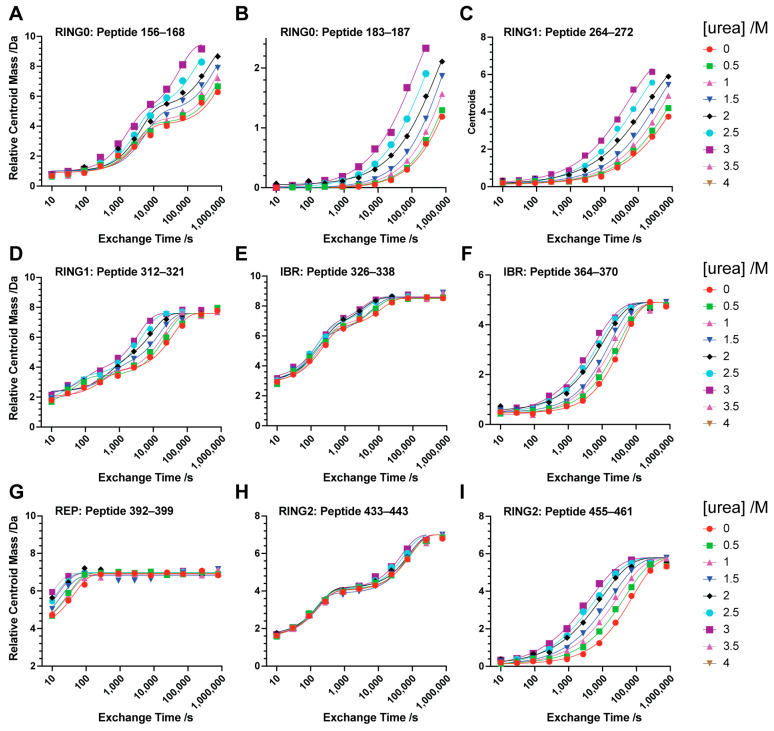
Structural fluctuations in Parkin(∆Ubl). Native-state hydrogen exchange time profiles representative of the various domains of Parkin(∆Ubl) measured by peptide fragmentation mass spectrometry (**A**–**I**). The domain and primary sequence residue numbers are shown in each panel. Fitting of these data (solid lines) gave average R^2^ and Sy.x values of 0.99 ± 0.02 and 0.16 ± 0.09 amu, respectively.

**Figure 4:**
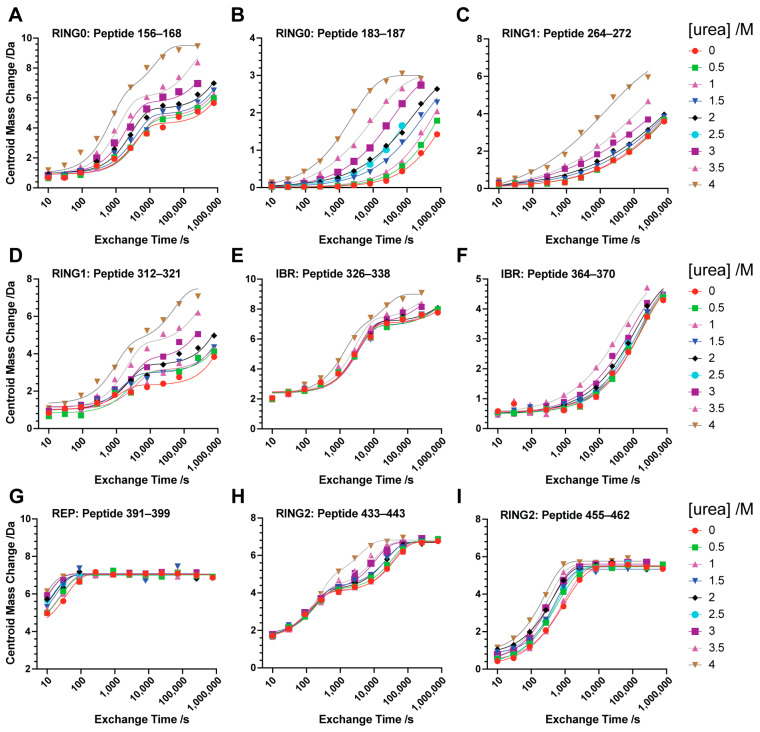
Structural fluctuations in the Parkin(∆Ubl)·pUb complex. Native-state hydrogen exchange time profiles representative of the various domains of Parkin(∆Ubl) in the Parkin(∆Ubl)·pUb complex measured by peptide fragmentation mass spectrometry (**A**–**I**). The domain and primary sequence residue numbers are shown in each panel. Fitting of these data (solid lines) gave average R^2^ and Sy.x values of 0.98 ± 0.02 and 0.16 ± 0.10 amu, respectively.

The high quality of H/D exchange (HX) data obtained is illustrated by representative time courses of peptides derived from the various globular domains of Parkin(∆Ubl) alone (Figure 3) and in complex with phosphorylated ubiquitin (pUb) (Figure 4). The fits of utilized HX time trajectories to the appropriate equation (see Methods) generally gave R^2^ values greater than 0.96 and root mean square error (RMSE) and Sy.x values less than 0.1 and 0.3 amu, respectively. Many of the time courses of HX within a given peptide show simple single exponential exchange patterns, while others exhibit multiple exponential exchange profiles. The extent of peptide coverage limits the analysis to small peptide sequences rather than nearly a single-amino-acid level of resolution.

The more complex time courses indicates that the amide hydrogens of two or more groups of residues within the peptide exchange hydrogen at different rates. Exchange of backbone amide NH with solvent is typically interpreted as an equilibrium between states where the amide hydrogen is protected from exchange and those where the NH is in contact with solvent water and available for exchange (termed the “closed” and “open” states, respectively) [24]. When the rate of reprotection (closing) is faster than the rate of chemical exchange of the unprotected NH with hydrogens of water, the ratio of the observed and intrinsic (unprotected) [21] hydrogen exchange rates gives an effective equilibrium constant (K_op_) and a corresponding “protection factor” (1/K_op_). The apparent free energy (∆G_HX_) of the structural transition(s) giving rise to unprotected states leading to exchange is then simply ∆G_HX_ = −RT ln(K_op_). This hydrogen exchange regime is expected under the conditions used here (pH 7.0 and 20 °C).

**Figure 5:**
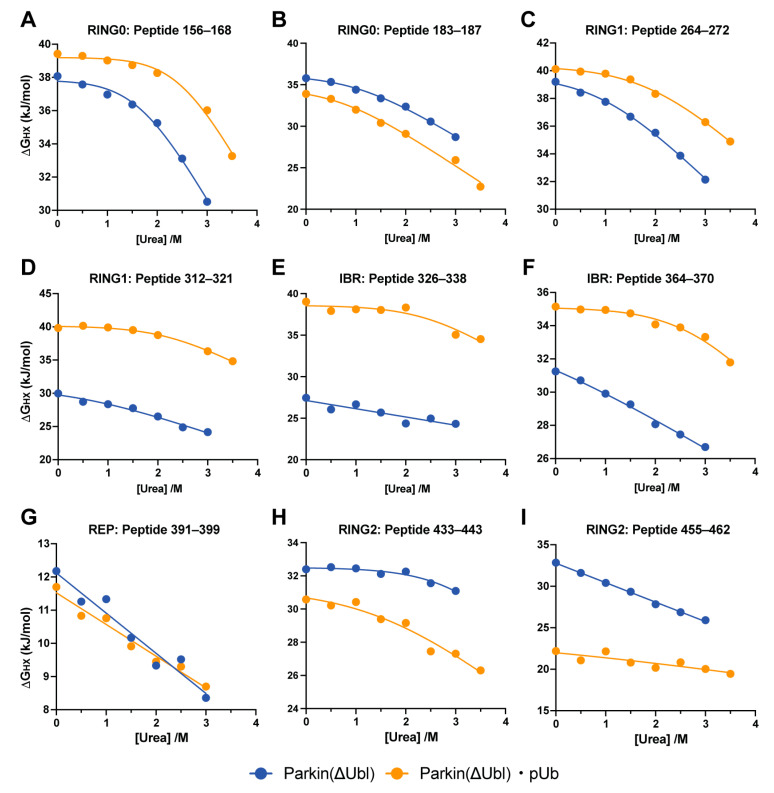
Comparison of the energetics of the protein ensemble revealed by native-state hydrogen exchange ${∆G}_{HX}^{dep}$ in Parkin(∆Ubl) and the Parkin(∆Ubl)·pUb complex. Denaturant dependence of the apparent free energy of hydrogen exchange (∆G_HX_) of representative peptides derived from the domains of Parkin(∆Ubl) (**A**–**I**). The domain and primary sequence residue numbers are shown in each panel. fitted with one or two exponentials as statistically warranted (see Table 1). Fitting of these data (solid lines) gave average R^2^ and Sy.x values of 0.96 ± 0.05 and 0.32 ± 0.17 kJ mol^−1^, respectively.

Fitting of the dependence of HX rates on urea concentration was similarly impressive. The urea dependence of the apparent free energy of hydrogen exchange (∆G_HX_) in the various domains of Parkin(∆Ubl) is shown in Figure 5. All five domains are represented. Generally, the curves are well fitted with linear terms representing denaturant-independent (m ~ 0) and denaturant-dependent (m > 0) exchange processes. In some cases, only a single term describing a urea-dependent exchange process was statistically warranted (e.g., Figure 4I). The fitted values ${∆G}_{HX}^{dep}$ for urea-dependent exchange in Parkin(∆Ubl) range from 46 kJ/mol to 12 kJ/mol and those of the complex with pUb range from 60 kJ/mol to 12 kJ/mol for the RING0 and REP domains, respectively (Table 1). The variation of ${∆G}_{HX}^{dep}$ for each domain suggests that Parkin(∆Ubl) does not unfold in a globally cooperative two-state manner. Rather, the individual domains unfold independently enough for this to be the dominant mechanism through which HX occurs. This is consistent with the averaged unfolding behavior seen for urea denaturation followed by CD spectroscopy (Figure 2).

The Parkin(∆Ubl)·pUb complex also gave excellent fits of the urea dependence of ${∆G}_{HX}$. As for free Parkin(∆Ubl), representative peptide sequences of some domains are minimally influenced by increasing urea (e.g., Figure 4E,I) while others show significant urea dependence (e.g., Figure 4B,D). Representative peptides of several domains show a double exponential time course like that seen for free Parkin(∆Ubl) (compare Panels A, D and H of Figure 3 and Figure 4). A common feature of these probe peptide sequences is the presence of zinc finger structural elements. Zinc fingers can have a remarkable affinity for divalent cations [29]. Clearly, this affinity stabilizes the protected (H-bonded) amide NH, and the involved structural elements are likely the last to be denatured by urea. Curiously, peptides 326–338 of the IBR domain transition to a clear double exponential time course with binding of pUb to Parkin(∆Ubl), suggesting that the zinc finger involving residues C332, C337, C360 and C352 has lower affinity for zinc in Parkin(∆Ubl) that is heightened in the Parkin(∆Ubl)·pUb complex through the allosteric effects of pUb binding.

### 3.3. Binding of the Allosteric Activator pUb to Parkin(∆Ubl) Has Heterogenous Effects

The binding of pUb to Parkin(∆Ubl) alters the apparent stability of all domains of Parkin(∆Ubl), but to differing degrees and in different directions (Figure 5 and Table 1). The RING1 and IBR domains, which have contacts with pUb in the complex, are stabilized to roughly the same extent (~20 kJ/mol). RING0 is nominally stabilized, but the beta sheet of RING0 holding the loop interacting with bound pUb has a mixed response and does not behave as a uniform cooperative unit. The apparent stability of the REP domain is unaffected by the binding of pUb. In contrast, the RING2 domain, containing the catalytic active site, is relatively strongly destabilized by the binding of pUb even though it is on the other side of the protein. The destabilization is, within error, uniform across the domain (Table 1).

**Figure 6:**
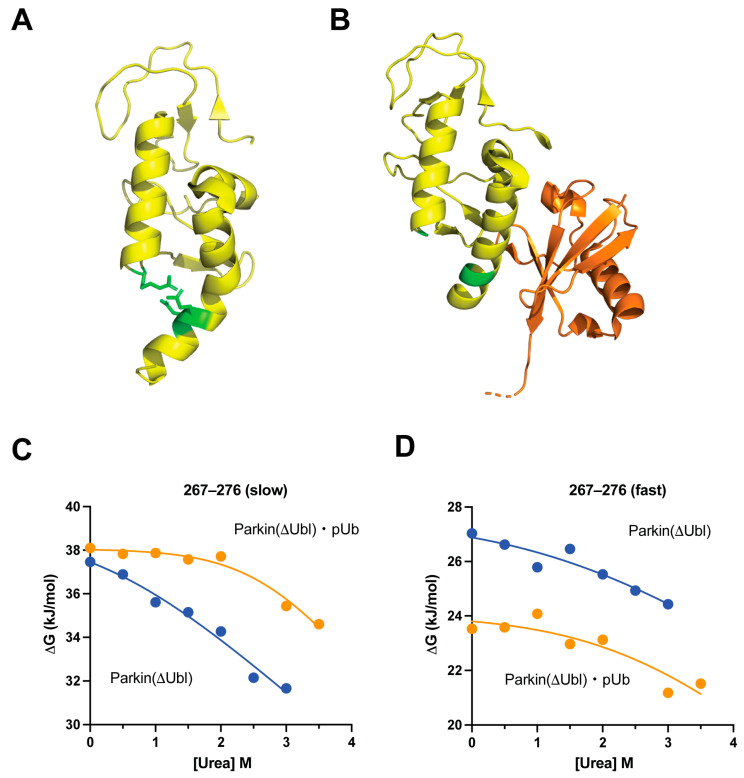
Allosteric transition in Parkin(∆Ubl) upon binding of pUb. Cartoon representations of the structure of the RING1 domain (shown in yellow) of (**A**) *hs*Parkin(∆Ubl) (PDB code: 4bm9) and (**B**) the *Ph*Parkin(∆Ubl)·pUb complex (PDB code: 5caw). pUb is colored orange. Shown in green are glutamate and arginine residues participating in an ion pair in *hs*Parkin(∆Ubl), which is disrupted in the *Ph*Parkin(∆Ubl)·pUb complex. Note that the involved E and R side chains are ill defined (disordered) in the *Ph*Parkin(∆Ubl))·pUb complex. The urea dependence of the apparent free energy of the fluctuations leading to hydrogen exchange in the helix spanning residues 268–280 in *hs*Parkin(∆Ubl) (**C**) and in the homologous region in the *Ph*Parkin(∆Ubl)·pUb complex (**D**). The HX time courses of this region in both the free and complexed Parkin(∆Ubl) are best fitted with two exponentials. Fitting of these data gave average R^2^ and Sy.x values of 0.94 ± 0.06 and 0.36 ± 0.11 kJ mol^−1^, respectively. Deconvolution using overlapping peptides indicates that the fast phase is associated with the C-terminus of this helix while the slower phase is associated with the N-terminal region. The C-terminus is destabilized by the binding of pUb. In contrast, the N-terminal region is stabilized by the binding of pUb. Cartoons drawn with PyMol.

Local structure at the interface of Parkin(∆Ubl) with the repressor effector Ubl domain, which acts in *cis*, is destabilized by the binding of the activating effector pUb even though they associate on opposite sides the RING1 domain. An interesting structural transition in the RING1 domain occurs upon binding of pUb—a deformed helix (residues 309–327) is regularized upon pUb binding [10]. The latter structure was determined using the highly homologous protein from *Pediculus humanus corporis* (*Ph*Parkin(∆Ubl)). The straightening of the helix is accompanied by breakage of a salt bridge cluster between E321 and E322 and R275 (Figure 6A,B). Hydrogen exchange in the adjacent helix spanning residues 261–276 (*Hs*Parkin) is complex, with reported peptides showing double exponential exchange profiles (Figure 6). Comparing the HX behavior of peptides reported on the helical segment spanning 268–280 indicates that the C-terminal end is destabilized while the N-terminal side is stabilized by the binding of pUb (Figure 6 C,D). Destabilization of the C-terminus of the helix is likely arising from increased helical end-fraying facilitated by the loss of the ion pairing of R275 (R277) with E321 (E323) in *Hs*Parkin (*Ph*Parkin). Conversely, binding of pUb stabilizes the N-terminal region of this helix against HX.

### 3.4. Dissociation of pUb from Parkin(∆Ubl)

The binding of pUb to Parkin(∆Ubl) also perturbs its own hydrogen exchange behavior. Though overall hydrogen exchange in pUb is minimally perturbed upon binding, the C-terminal β-strand offers an interesting insight into the mechanism of molecular recognition. Representative peptides spanning residues 60 to 72 show unremarkable *m*/*z* profiles as a function of time of hydrogen exchange (Figure S1A). In contrast, early time points in the hydrogen exchange experiment with the Parkin(∆Ubl)·pUb complex show the existence of two populations. The experiment was carried out with a 2:1 pUb-to-Parkin(∆Ubl) ratio. One population exchanges in the EX2-manner (i.e., its mass smoothly increases) and at a rate that is characteristic of free pUb. The other shows much slower EX2-type exchange but has a faster rate of disappearance. This is perhaps reminiscent of EX1-type HX behavior, where the rate of reprotection of exchange-competent states is slower than the intrinsic chemical rate of exchange. However, this is ruled out by the initial EX2 behavior. A simple explanation is that when pUb dissociates from Parkin(∆Ubl), it is diluted into the excess free pUb, and thus, the exchange is dominated by the faster free pUb. As a result, the lower *m*/*z* population disappears and reappears in the higher *m*/*z* population. Free pUb continues to exchange amide hydrogens with solvent via the EX2 mechanism, and so the population moves to higher *m*/*z* as HX proceeds to completion.

Reported hydrogen exchange by eight overlapping peptides spanning residues 60–72 in pUb could be quantified. The *m*/*z* time courses could be fitted well will a simple single exponential curve (Figure S1C). The rate of dissociation of pUb from Parkin(∆Ubl) was obtained from the time courses of the slowly exchanging population reported by these peptides and averaged. This rate as a function of urea concentration was measured. The obtained m-value of 0.83 ± 0.11 kJ mol^−1^ M^−1^ is surprisingly small given the extent of the surface area comprising the Parkin(∆Ubl)·pUbl interface.

## 4. Discussion

E3 ubiquitin ligases are deeply involved in the manifestation of neurological disorders [30,31]. Parkin is one such example where both regulatory dysfunction and corruption of proteostasis result from disease-causing mutations. Over the past decade or so, there has been remarkable progress in our understanding of the structural underpinnings of the regulatory control of Parkin [9,13,14,25,32,33,34,35,36]. These studies have illuminated the complexity of allosteric regulation of the enzyme where the antagonist Ubl domain, attached via a disordered linker and acting in *cis*, competes with the agonist pUb binding in *trans*. Layered on this primary regulatory lever is phosphorylation of the Ubl domain, promoting its dissociation from its inhibitory site on the Parkin core and transit to an alternate location, presumably with activating properties [14]. In addition, the binding of the E2~substrate to the REP domain likely modulates the influence of the allosteric effectors and post-translational modifications of the protein. Clearly, disentangling the energetics of the various interactions involved in this complex regulatory scenario is required before a fundamental understanding of the influence of disease-causing missense mutations can be reached.

Herein, we have begun with an examination of a version of Parkin where the Ubl domain and linker sequence have been deleted, i.e., Parkin(∆Ubl), and its interaction with the agonist pUb. We have employed hydrogen exchange monitored by peptide fragmentation mass spectrometry [37]. Hydrogen exchange methodology has been employed previously to detect changes in Parkin structure that result in (de)protection of amide NH [13,25]. However, extracting detailed information regarding the energetics of structural transitions associated with allosteric regulation from changes in simple hydrogen exchange rates is confounded by the contribution of different physical mechanisms that can lead to deprotection of otherwise H-bonded amide NH. Commonly, the ranking of observed hydrogen exchange rates does not correspond to the ranking of simple stability. The so-called native-state hydrogen exchange approach seeks to tease apart the various contributions to hydrogen exchange at a given residue by relying on the differential changes in the accessible surface upon local, subglobal and global unfolding of the protein as probed by a chemical denaturant [15].

### 4.1. Cooperative Substructure of the Parkin Core and the Allosteric Response

In many proteins, the native-state HX approach has revealed the presence of discrete cooperative units of structure, commonly termed “foldons” [23]. A striking observation here is that a simple urea denaturation profile reveals that Parkin(∆Ubl) is remarkably uncooperative with respect to global unfolding. This is reflected in the apparent free energy profiles of the various domains of Parkin(∆Ubl). The variation in ∆G_HX_ across the five domains indicates that there is not a global cooperative unfolding event. Rather, much more frequent so-called subglobal but cooperative unfolding within the individual domains is indicated. For example, reporter peptides within the catalytic RING2 structural domain indicate that it is composed of at least two energetically distinct cooperative units. The same is true of the RING0 and RING1 structural domains. Reporter peptides for the IBR domain potentially suggest that it behaves as single cooperative unit of structure. Importantly, the hydrogen exchange view indicates that the most stable elements of the protein have ${∆G}_{unf}\geq$ 60 kJ mol^−1^ (Table 1).

The binding of the allosteric agonist pUb to Parkin(∆Ubl) results in significant changes in ∆G_HX_ of reporters in the RING2 and RING1 domains. It is important to note that the resulting values of ∆∆G_HX_ are, within error, uniform within each of these domains but of different magnitude and of opposite sign. The RING1 domain is stabilized against hydrogen exchange. In other words, binding of the pUb agonist stabilizes the transition of the RING1 domain towards a structure that is less compatible with association with the antagonist Ubl domain. The apparent free energy change associated with this transition (∆∆G_HX_ = −18.8 ± 3.3 kJ/mol) is large and favorable. In contrast, binding of pUb destabilizes the RING2 domain against hydrogen exchange. The uniformity of the allosteric response in the RING2 domain (∆∆G_HX_ = +9.6 ± 3.6 kJ/mol) suggests a simple explanation: the dominant contribution to the observed hydrogen exchange arises from detachment of the entire RING2 domain from the remainder of the core. The destabilization results in enhanced deprotection and an overall reduction in exposed surface area consistent with the lower m-values. In this interpretation, the binding of pUb therefore pushes the equilibrium to the detached state by ~50-fold. It is from this detached state that RING2 reorganizes to expose the active site of the ligase to the E2~protein substrate [14]. More subtle effects of the binding of pUb are also seen. For example, the zinc finger involving residues C332, C337, C360 and C352 has relatively low affinity for zinc in Parkin(∆Ubl) with urea denaturation, but the affinity is heightened in the Parkin(∆Ubl)·pUb complex through the allosteric effects of pUb binding. In a manner reminiscent of the oxy/deoxyhemoglobin transition, the disruption of an ionic interaction stabilizing the repressed state of the Parkin core is disrupted by the binding of pUb. Similarly, this allosteric transition, like that of hemoglobin [38], perturbs the manifold of states visited by this region of the protein.

**Figure 7:**
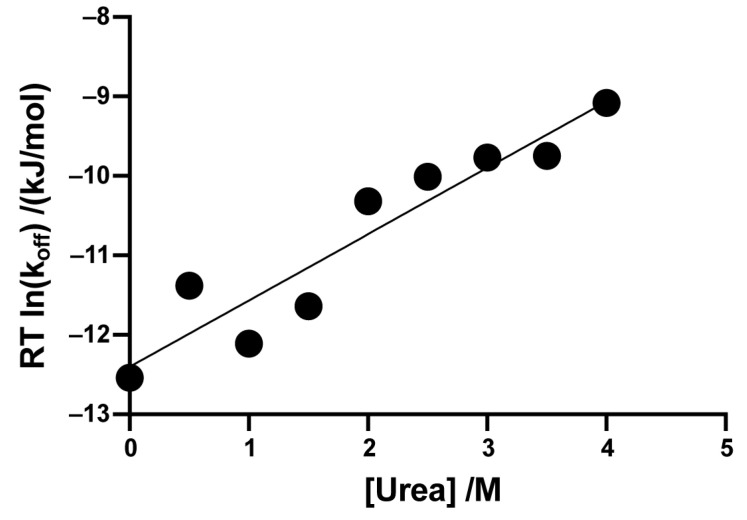
Urea dependence of the activation free energy of dissociation of pUb from Parkin(∆Ubl). Rates of dissociation of pUb from Parkin(∆Ubl) were extracted from two population binomial fits of the *m*/*z* distribution HX time courses of the Parkin(∆Ubl)·pUb complex as a function of urea (see Figure S1). The fitted m-value was 0.83 ± 0.11 kJ mol^−1^ M^−1^ and R^2^ 0.89.

The dependence of the rate of dissociation on urea concentration presents insight into the transition state (Figure 7). The dependence of pUb dissociation rate on urea concentration is rather weak (0.83 ± 0.11 kJ mol^−1^ M^−1^). The low cooperativity of global unfolding of the protein and the uncertainty of the structure of transition state somewhat limit the interpretation of the *m*-value in this context. If the transition state barrier to dissociation corresponds to simple separation and solvation of the interface that is followed by energetically downhill structural transition, then the large surface area exposed upon dissociation of pUb (~2700 Å^2^) would suggest a larger m-value than that observed. It is noted that the dissociation of the complex does not greatly perturb the secondary structure of pUb, ruling it out as the primary mechanism for urea-induced structure change [39,40,41]. The interface itself is a complex mixture of side chains with both stabilizing and destabilizing interactions with urea [40]. Nevertheless, the relatively low *m*-value suggests that only subtle organization of the complex is required to reach the transition state for dissociation of pUb.

### 4.2. Stability, Molecular Recognition and Disease-Causing Mutations

Loss-of-function mutations in the PRKN gene account for about 50% of autosomal recessive juvenile Parkinsonism and three-quarters of familial early-onset Parkinson’s disease [2,4]. The causal relationships between missense mutations and specific aspects of Parkin’s function are complicated, and many remain obscure [42,43]. The reciprocal allosteric relationship of the free energy of binding of pUb and the destabilization of the binding interface with the antagonist Ubl domain and the interface with the catalytic RING2 domain as found here not only illuminates the allosteric mechanism but also rationalizes some mutations leading to hyperactivity (see Stevens et al. [44]). At a more fundamental level, efforts to understand the effects of missense mutations have increasingly focused on their impact on protein thermodynamic and biochemical stability or proteostasis [45,46,47]. Indeed, a saturation mutational analysis of steady-state cellular abundance of Parkin found that that most low-abundance variants are proteasome targets and are located within structured domains, i.e., they are consistent with destabilization of the protein [3]. Half of the known disease-linked variants are found at low abundance [3]. There now appears to be a strong relationship between cellular abundance levels and likely exposure of degron signals due to stabilization of globular structure by missense mutations [45]. The complex set of stabilities of apparent foldons within the structure of Parkin is consistent with the impact of disease-causing missense mutations across the primary sequence. In other words, in this view, the fact that the core of Parkin is composed of subglobal units of cooperative structure renders it sensitive to fluctuations that expose it to proteasome targeting.

## 5. Conclusions

Tremendous advances over the past decade in the structural biology of Parkin have provided a foundation for understanding its catalytic action and allosteric regulation. Formation and destruction of hydrogen bonding, monitored by “snapshot” hydrogen deuterium exchange studies, has confirmed many of the details of the structural transitions leading to activation. Application of the native-state HX strategy herein has enabled the dissection of the stability and cooperative structure of the Parkin core. In this context, the energetics of the allosteric regulation by the activating effector pUb has been revealed. A reciprocal relationship between the binding of pUb and the stability of the interface that is formed with the antagonist Ubl domain and the inactive state of the catalytic domain provides a thermodynamic platform for future studies of disease-causing Parkin mutants.

## Figures and Tables

**Table 1:** Native-state hydrogen exchange in Parkin(∆Ubl) and the Parkin(∆Ubl))·pUb complex ^1^.

		Parkin (∆Ubl)			Parkin (∆Ubl)·pUb			
Domain	Peptide	${∆G}_{HX}^{ind}$ kJ mol	${∆G}_{HX}^{dep}$ kJ/mol	$m^{dep}$ kJ/mol/M	${∆G}_{HX}^{ind}$ kJ mol	${∆G}_{HX}^{dep}$ kJ/mol	$m^{dep}$ kJ/mol/M	${∆∆G}_{HX}^{dep}$ kJ/mol
RING0	156–168	37.8 ± 0.2	46.4 ± 1.1	5.2 ± 0.4	39.2 ± 0.2	52.5 ± 2.5	5.4 ± 0.8	−6.1 ± 2.7
	183–187	36.2 ± 0.2	40.0 ± 0.6	3.7 ± 0.2	34.6 ± 1.4	37.2 ± 1.0	4.0 ± 0.3	+2.8 ± 1.2
RING1	264–272	38.6 ± 0.3	39.7 ± 0.2	6.3 ± 0.1	37.7 ± 0.1	59.5 ± 3.0	7.6 ± 0.9	−19.8 ± 3.0
	312–321	32.0 ± 0.3 *	31.0 ± 1.1	2.6 ± 0.4	40.1 ± 0.1	48.8 ± 0.9	3.8 ± 0.3	−17.8 ± 1.4
IBR	326–338	n.s.	27.1 ± n.d.	1.0 ± n.d.	37.6 ± n.d.	48.0 ± 4.9	3.8 ± 1.5	−20.9 ± n.d.
	364–370	n.s.	31.4 ± 0.1	1.6 ± 0.1	35.1 ± 0.5	44.4 ± 1.8	3.3 ± 0.6	−13.0 ± 1.8
REP	391–399	n.s.	12.2 ± 0.2	1.2 ± 0.1	n.s.	11.5 ± 0.1	1.0 ± 0.1	+0.7 ± 0.2
RING2	433–443	32.5 ± 1.7	43.4 ± 3.3	3.5 ± 1.0	31.2 ± 0.2 *	34.6 ± 1.5	2.3 ± 0.5	+8.8 ± 3.6
	455–462	n.s.	32.8 ± 0.1	2.3 ± 0.1	n.s.	22.5 ± 0.3	0.7 ± 0.2	+10.3 ± 0.3

^1^ See main text for definition of native-state HX parameters ${∆G}_{HX}^{ind}$, ${∆G}_{HX}^{dep}$ and $m^{dep}$; ${{∆∆G}_{HX}^{dep}=∆G}_{HX}^{dep}$ (Parkin(∆Ubl)) − ${∆G}_{HX}^{dep}$ (Parkin(∆Ubl)·pUb) ; n.s., not statistically significant; n.d., not determined, unstable solution; *, approximated.

## Data Availability

Simple text files of primary *m*/*z* distributions for all quantifiable peptides of acceptable quality are deposited in the Texas Data Repository (https://doi.org/10.18738/T8/HS8M9X accessed on 16 October 2024).

## Supplementary Materials

The following supporting information can be downloaded at: https://www.mdpi.com/article/10.3390/biom14101338/s1. Figure S1: Precision of HX-MS experiments. Figures S2–S4: Statistics of peptide fragmentation. Figure S5: Hydrogen exchange in free and complexed pUb.

## References

[1] Um J.H., Yun J. (2017). Emerging role of mitophagy in human diseases and physiology. BMB Rep..

[2] Lucking C.B., Durr A., Bonifati V., Vaughan J., De Michele G., Gasser T., Harhangi B.S., Meco G., Denefle P., Wood N.W. (2000). Association between early-onset Parkinson’s disease and mutations in the parkin gene. N. Engl. J. Med..

[3] Clausen L., Voutsinos V., Cagiada M., Johansson K.E., Gronbaek-Thygesen M., Nariya S., Powell R.L., Have M.K.N., Oestergaard V.H., Stein A. (2024). A mutational atlas for Parkin proteostasis. Nat. Commun..

[4] Kitada T., Asakawa S., Hattori N., Matsumine H., Yamamura Y., Minoshima S., Yokochi M., Mizuno Y., Shimizu N. (1998). Mutations in the parkin gene cause autosomal recessive juvenile parkinsonism. Nature.

[5] Pickrell A.M., Youle R.J. (2015). The roles of PINK1, Parkin, and mitochondrial fidelity in Parkinson’s disease. Neuron.

[6] Shimura H., Hattori N., Kubo S., Mizuno Y., Asakawa S., Minoshima S., Shimizu N., Iwai K., Chiba T., Tanaka K. (2000). Familial Parkinson disease gene product, parkin, is a ubiquitin-protein ligase. Nat. Genet..

[7] Hedrich K., Eskelson C., Wilmot B., Marder K., Harris J., Garrels J., Meija-Santana H., Vieregge P., Jacobs H., Bressman S.B. (2004). Distribution, type, and origin of Parkin mutations: Review and case studies. Mov. Disord..

[8] Cookson M.R. (2005). The biochemistry of Parkinson’s disease. Annu. Rev. Biochem..

[9] Trempe J.F., Sauve V., Grenier K., Seirafi M., Tang M.Y., Menade M., Al-Abdul-Wahid S., Krett J., Wong K., Kozlov G. (2013). Structure of Parkin reveals mechanisms for ubiquitin ligase activation. Science.

[10] Wauer T., Simicek M., Schubert A., Komander D. (2015). Mechanism of phospho-ubiquitin-induced Parkin activation. Nature.

[11] Jumper J., Evans R., Pritzel A., Green T., Figurnov M., Ronneberger O., Tunyasuvunakool K., Bates R., Zídek A., Potapenko A. (2021). Highly accurate protein structure prediction with AlphaFold. Nature.

[12] Varadi M., Anyango S., Deshpande M., Nair S., Natassia C., Yordanova G., Yuan D., Stroe O., Wood G., Laydon A. (2022). AlphaFold Protein Structure Database: Massively expanding the structural coverage of protein-sequence space with high-accuracy models. Nucleic Acids Res..

[13] Gladkova C., Maslen S.L., Skehel J.M., Komander D. (2018). Mechanism of parkin activation by PINK1. Nature.

[14] Trempe J.F., Gehring K. (2023). Structural Mechanisms of Mitochondrial Quality Control Mediated by PINK1 and Parkin. J. Mol. Biol..

[15] Bai Y.W., Sosnick T.R., Mayne L., Englander S.W. (1995). Protein folding intermediates—Native state hydrogen exchange. Science.

[16] Pan M., Zheng Q.Y., Gao S., Qu Q., Yu Y.Y., Wu M., Lan H., Li Y.L., Liu S.L., Li J.B. (2019). Chemical synthesis of structurally defined phosphorylated ubiquitins suggests impaired Parkin activation by phosphorylated ubiquitins with a non-phosphorylated distal unit. CCS Chem..

[17] Santoro M.M., Bolen D.W. (1988). Unfolding free energy changes determined by the linear extrapolation method. 1. Unfolding of phenylmethanesulfonyl a-chymotrypsin using different denaturants. Biochemistry.

[18] Pace C.N., Shaw K.L. (2000). Linear extrapolation method of analyzing solvent denaturation curves. Proteins.

[19] Mayne L., Kan Z.Y., Chetty P.S., Ricciuti A., Walters B.T., Englander S.W. (2011). Many overlapping peptides for protein hydrogen exchange experiments by the fragment separation-mass spectrometry method. J. Am. Soc. Mass Spec..

[20] Kan Z.Y., Ye X., Skinner J.J., Mayne L., Englander S.W. (2019). ExMS2: An integrated solution for hydrogen-deuterium exchange mass spectrometry data analysis. Anal. Chem..

[21] Bai Y.W., Milne J.S., Mayne L., Englander S.W. (1993). Primary structure effects on peptide group hydrogen exchange. Proteins.

[22] Krissinel E., Henrick K. (2007). Inference of macromolecular assemblies from crystalline state. J. Mol. Biol..

[23] Englander S.W. (2023). HX and Me: Understanding allostery, folding, and protein machines. Annu. Rev. Biochem..

[24] Englander S.W., Kallenbach N.R. (1983). Hydrogen exchange and structural dynamics of proteins and nucleic acids. Q. Rev. Biophys..

[25] Sauve V., Sung G., Soya N., Kozlov G., Blaimschein N., Miotto L.S., Trempe J.F., Lukacs G.L., Gehring K. (2018). Mechanism of parkin activation by phosphorylation. Nat. Struct. Mol. Biol..

[26] Fuentes E.J., Wand A.J. (1998). Local stability and dynamics of apocytochrome b(562) examined by the dependence of hydrogen exchange on hydrostatic pressure. Biochemistry.

[27] Kranz J.K., Flynn P.F., Fuentes E.J., Wand A.J. (2002). Dissection of the pathway of molecular recognition by calmodulin. Biochemistry.

[28] Chen Y.H., Yang J.T., Martinez H.M. (1972). Determination of secondary structures of proteins by circular dichroism and optical rotary dispersion. Biochemistry.

[29] Kluska K., Adamczyk J., Krezel A. (2018). Metal binding properties, stability and reactivity of zinc fingers. Coord. Chem. Rev..

[30] George A.J., Hoffiz Y.C., Charles A.J., Zhu Y., Mabb A.M. (2018). A comprehensive atlas of E3 ubiquitin ligase mutations in neurological disorders. Front. Genet..

[31] Ahel J., Lehner A., Vogel A., Schleiffer A., Meinhart A., Haselbach D., Clausen T. (2020). Moyamoya disease factor RNF213 is a giant E3 ligase with a dynein-like core and a distinct ubiquitin-transfer mechanism. Elife.

[32] Beasley S.A., Hristova V.A., Shaw G.S. (2007). Structure of the Parkin in-between-ring domain provides insights for E3-ligase dysfunction in autosomal recessive Parkinson’s disease. Proc. Natl. Acad. Sci. USA.

[33] Chaugule V.K., Burchell L., Barber K.R., Sidhu A., Leslie S.J., Shaw G.S., Walden H. (2011). Autoregulation of Parkin activity through its ubiquitin-like domain. EMBO J..

[34] Wauer T., Komander D. (2013). Structure of the human Parkin ligase domain in an autoinhibited state. EMBO J..

[35] Kumar A., Aguirre J.D., Condos T.E.C., Martinez-Torres R.J., Chaugule V.K., Toth R., Sundaramoorthy R., Mercier P., Knebel A., Spratt D.E. (2015). Disruption of the autoinhibited state primes the E3 ligase parkin for activation and catalysis. EMBO J.

[36] Sauve V., Lilov A., Seirafi M., Vranas M., Rasool S., Kozlov G., Sprules T., Wang J., Trempe J.F., Gehring K. (2015). A Ubl/ubiquitin switch in the activation of Parkin. EMBO J..

[37] Kan Z.Y., Walters B.T., Mayne L., Englander S.W. (2013). Protein hydrogen exchange at residue resolution by proteolytic fragmentation mass spectrometry analysis. Proc. Natl. Acad. Sci. USA.

[38] Englander S.W., Englander J.J., McKinnie R.E., Ackers G.K., Turner G.J., Westrick J.A., Gill S.J. (1992). Hydrogen-exchange measurement of the free-energy of structural and allosteric change in hemoglobin. Science.

[39] Lim W.K., Rösgen J., Englander S.W. (2009). Urea, but not guanidinium, destabilizes proteins by forming hydrogen bonds to the peptide group. Proc. Natl. Acad. Sci. USA.

[40] Auton M., Holthauzen L.M.F., Bolen D.W. (2007). Anatomy of energetic changes accompanying urea-induced protein denaturation. Proc. Natl. Acad. Sci. USA.

[41] Hong J., Capp M.W., Saecker R.M., Record M.T. (2005). Use of urea and glycine betaine to quantify coupled folding and probe the burial of DNA phosphates in Lac repressor—Lac operator bindings. Biochemistry.

[42] Martin I., Dawson V.L., Dawson T.M. (2011). Recent advances in the genetics of Parkinson’s disease. Annu. Rev. Genom. Human Genet..

[43] Yi W., MacDougall E.J., Tang M.Y., Krahn A.I., Gan-Or Z., Trempe J.F., Fon E.A. (2019). The landscape of <Parkin variants reveals pathogenic mechanisms and therapeutic targets in Parkinson’s disease. Human Mol. Genetics.

[44] Stevens M.U., Croteau N., Eldeeb M.A., Antico O., Zeng Z.W., Toth R., Durcan T.M., Springer W., Fon E.A., Muqit M.M. (2023). Structure-based design and characterization of Parkin-activating mutations. Life Sci. Alliance.

[45] Stein A., Fowler D.M., Hartmann-Petersen R., Lindorff-Larsen K. (2019). Biophysical and mechanistic models for disease-causing protein variants. Trends Biochem. Sci..

[46] Kampmeyer C., Larsen-Ledet S., Wagnkilde M.R., Michelsen M., Iversen H.K.M., Nielsen S.V., Lindemose S., Caregnato A., Ravid T., Stein A. (2022). Disease-linked mutations cause exposure of a protein quality control degron. Structure.

[47] Cagiada M., Johansson K.E., Valanciute A., Nielsen S.V., Hartmann-Petersen R., Yang J.J., Fowler D.M., Stein A., Lindorff-Larsen K. (2021). Understanding the Origins of Loss of Protein Function by Analyzing the Effects of Thousands of Variants on Activity and Abundance. Mol. Biol. Evol..

