# Supplementary material for: Cooperative Substructure and Energetics of Allosteric Regulation of the Catalytic Core of the E3 Ubiquitin Ligase Parkin by Phosphorylated Ubiquitin
Source: Biomolecules. 2024 Oct 21;14(10):1338. doi: 10.3390/biom14101338 (PMC11506642; doi:10.3390/biom14101338)
Supplement: Supplementary file 1 [file biomolecules-14-01338-s001.zip › biomolecules-3192077-supplementary.pdf]

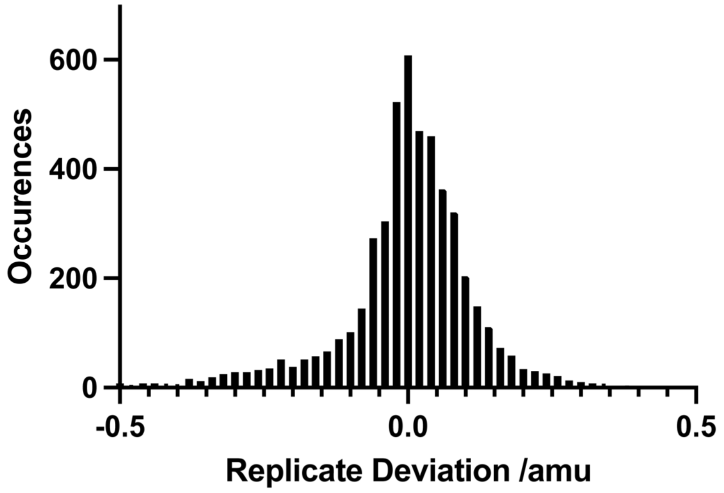

Figure S1. Precision of hydrogen exchange monitored by HX-MS in Parkin is high. Distribution of deviations of the fitted m/z centroid positions between two independently measured hydrogen exchange time courses. 4966 comparisons gave a mean of -0.003 and a root mean squared error of 0.18 amu.

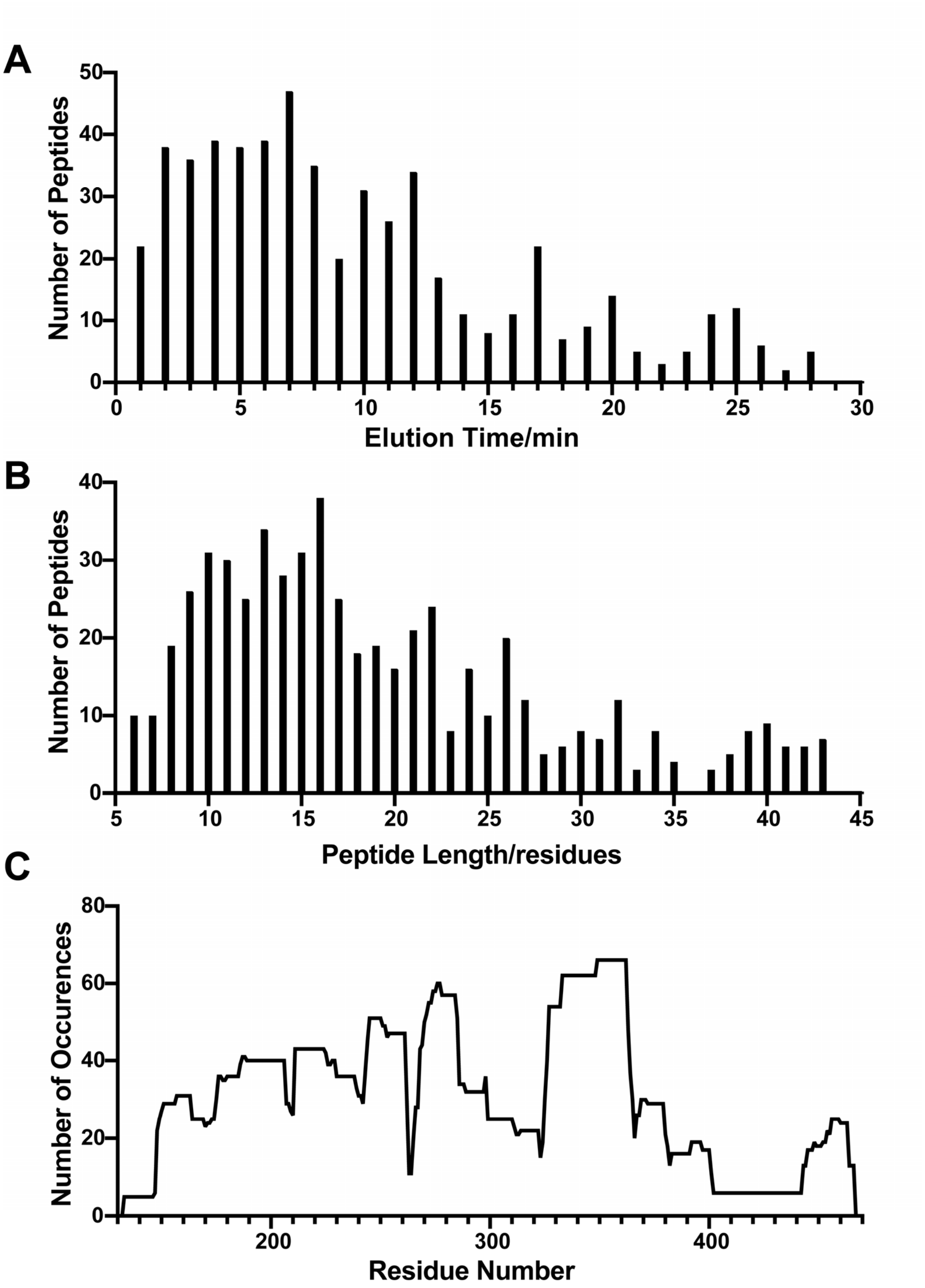

Figure S2. Summary of peptide fragmentation of free Parkin(ΔUbl). (A) Histogram of the reverse phase chromatography elution peptide elution profile. (B) Histogram summarizing the distribution of peptide sizes. (C) Sequence coverage of the peptide coverage at reach residue of the Parkin(ΔUbl) sequence.

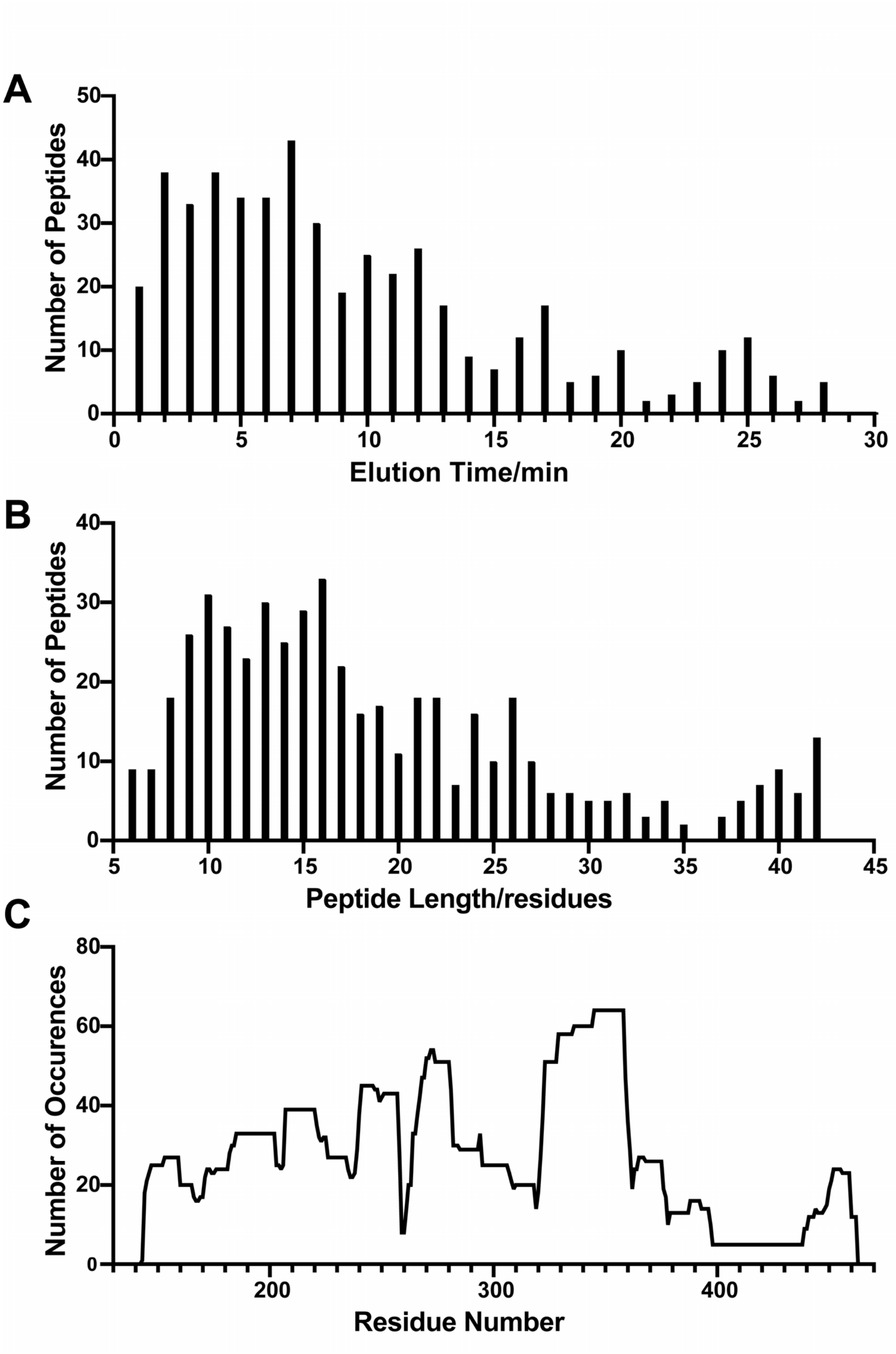

Figure S3. Summary of peptide fragmentation of Parkin(ΔUbl) from the Parkin(ΔUbl)•pUb complex. (A) Histogram of the reverse phase chromatography elution peptide elution profile. (B) Histogram summarizing the distribution of peptide sizes. (C) Sequence coverage of the peptide coverage at reach residue of the Parkin(ΔUbl) sequence.

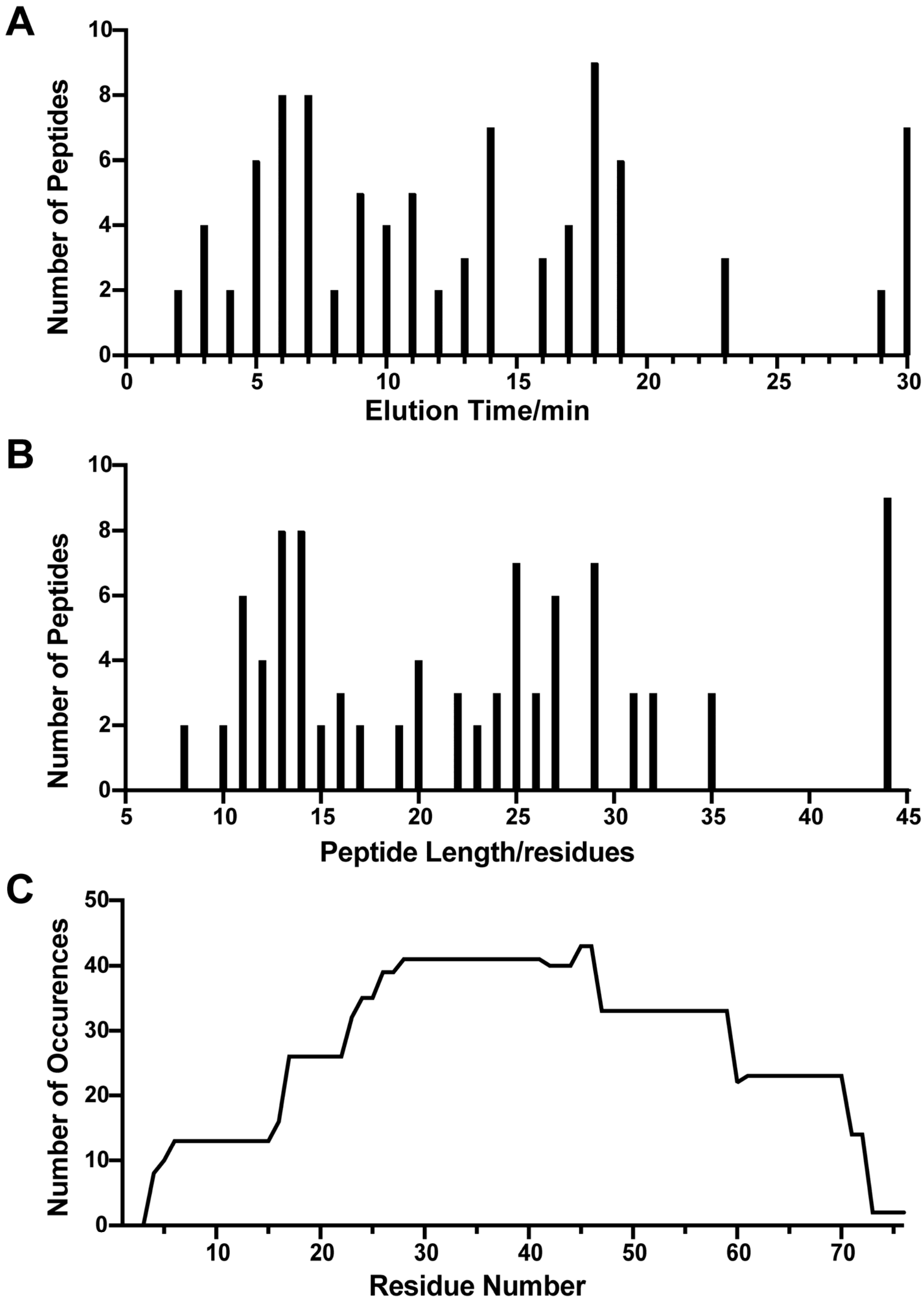

Figure S4. Summary of peptide fragmentation of pUb from the Parkin(ΔUbl)•pUb complex. (A) Histogram of the reverse phase chromatography elution peptide elution profile. (B) Histogram summarizing the distribution of peptide sizes. (C) Sequence coverage of the peptide coverage at reach residue of the Parkin(ΔUbl) sequence.

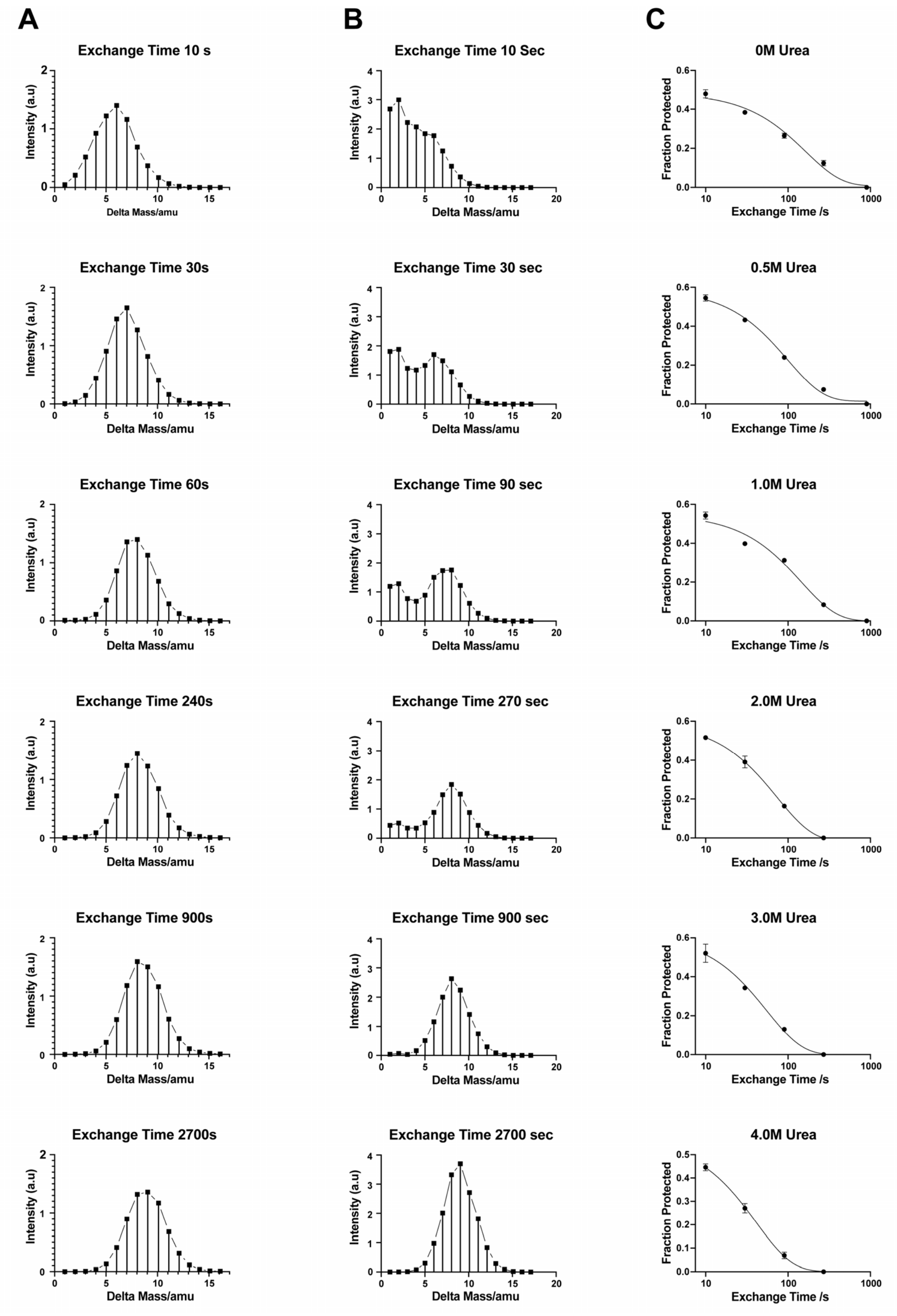

Figure S5. Hydrogen exchange in free and complexed pUb. Individual m/z distributions during hydrogen exchange derived from residues 60-72 in free (A) and complexed (B) pUb. Free pUb shows a smooth transition to higher mass indicative of EX2-type hydrogen exchange. pUb complexed to Parkin(ΔUbl) shows two distinct populations, one exchanging as free pUb and a slower exchanging population corresponding to complexed pUb. Note that pUb and Parkin(ΔUbl) were in a 2:1 molar ratio. (C) The fractional protection of the average of eight separate peptides derived from residues 60-72 of pUb were fitted to simple single exponential decays. Fitting of these data gave average R^2 and Sy.x values of 0.99 ± 0.01 and 0.02 ± 0.01, respectively.
